# Efficacy of Photobiomodulation Therapy in the Treatment of Pain and Inflammation: A Literature Review

**DOI:** 10.3390/healthcare11070938

**Published:** 2023-03-24

**Authors:** Ana González-Muñoz, María Cuevas-Cervera, José Javier Pérez-Montilla, Daniel Aguilar-Núñez, Dina Hamed-Hamed, María Aguilar-García, Leo Pruimboom, Santiago Navarro-Ledesma

**Affiliations:** 1 Department of Physiotherapy, Faculty of Health Sciences, Campus of Melilla, University of Granada, Querol Street 5, 52004 Melilla, Spain; 2 Clinica Ana Gonzalez, Avenida Hernan Nuñez de Toledo 6, 29018 Malaga, Spain; 3 Department of Nursing and Podiatry, Faculty of Health Sciences, University of Malaga, 29071 Malaga, Spain; 4 Department of Physiotherapy, University Chair in Clinical Psychoneuroimmunology (University of Granada and PNI Europe), 2518 JP The Hague, The Netherlands

**Keywords:** low-level laser therapy, photobiomodulation, chronic pain, inflammation, metabolism

## Abstract

The main objective of this literature review was to analyze the efficacy of (PBM) therapy application on subjects with chronic pain and inflammation, and furthermore, to evaluate the methodological quality of the collected literature. The search was conducted using five databases: PubMed, ProQuest, Scopus, Web of Science, and PEDro. The keywords “low level laser therapy”, “chronic pain”, and “inflammation” provided the selection of RCTs that were published within the last 5 years, conducted in humans, and written in English. The PEDro Internal Validity Scale (IVS) checklist was used to evaluate the risk of bias in the included studies. A total of 11 articles were selected, all of them RCTs. Of the articles, five showed that PBM positively influences chronic pain, while another showed the same but only in the short term. In two other articles, the patient’s inflammation improved markedly. In one article there was no improvement in chronic pain and in another, there was no improvement in inflammation. Four articles demonstrated that PBM is beneficial in acute pain. Furthermore, six studies were given an “excellent” score and the remaining five a “good” score based on the IVS. Photobiomodulation has beneficial effects on chronic pain and inflammation, although more research needs to be completed in this line for this to be clarified as the existence of RCTs on this subject is limited.

## 1. Introduction

Photobiomodulation therapy (PBM) commonly uses wavelengths of light with an energy density ranging from 1 to 150 J/cm^2^ and from 600 to 1070 nm. The effective tissue penetration is maximal in this range with hemoglobin and melanin, as the principal tissue chromophores, having high absorption bands at wavelengths shorter than 600 nm. The treatment of superficial tissue uses wavelengths in the range of 600 to 700 nm, while the treatment of deeper tissue uses wavelengths in the range of 780 to 950 nm [1,2]. Currently, whole-body PBM has shown a systemic response in addition to the local response, with improvements in quality of life, pain, sleep disorders, tiredness, muscle spasm, morning stiffness, psychological factors, elastic properties of tissue, circadian rhythms, tender points, and in fibromyalgia sufferers [3,4,5,6,7].

Furthermore, PBM therapy has also been shown to improve cerebral blood flow, neuronal bioenergetic functions, neuroinflammation, oxidative stress, neural apoptosis, neurogenesis, and neurotrophic factors, and additionally has effects on intrinsic brain networks [1]. In addition, PBM is also thought to affect the secretion of certain hormones, such as serotonin and endorphins, leading to a reduction in pain signaling [8].

Chronic pain is a problem that has a very notorious impact on society and people’s lives and is one of the most common health problems among older adults (>65 years). It is estimated that 13–50% of adults in the United Kingdom suffer from chronic pain although it is difficult to obtain accurate data since the estimates for the prevalence in the population vary greatly by place, time, and population. It has been estimated to be 8% per year in the UK. On the other hand, the US sets its estimated costs attributable to chronic pain, including disability, loss of work, and treatments, at about USD 600 million annually, with an incidence of 28.4% in the adult population. Treatment in these older adults is complex since it must have a multifactorial focus that includes pharmacological interventions, physical rehabilitation, and procedures to eradicate the cycle of pain [9,10,11,12]. By definition, chronic pain is pain that persists for at least 3 months. It is a factor in premature death and accelerated cognitive deterioration. In turn, this deterioration and dementia make treatment decisions difficult since the patient’s ability to perceive pain and report it is impaired [11].

Inflammation is present in many chronic pain conditions and its reduction is usually related to a decrease in pain. Oxidative stress is known to contribute to this inflammation [12]. Prolonged inflammation can lead to chronic hypersensitivity and perpetuate chronic pain. Several factors have been identified that influence inflammation induced by oxidative stress, chemokines, transcription factors, microRNAs, and inflammatory cytokines [12]. It has also been proven that, if widespread inflammation in neurological tissue, which has been caused by a failure of the immune system to promote homeostasis, lasts beyond a certain time, it can result in the onset of chronic pain. When this failure continues, low-grade inflammation (LGI) and existing damage develop, and its perpetuation indicates the development of chronic musculoskeletal pain, in addition to peripheral hypersensitization, which evolves into central hypersensitization. Therefore, LGI is thought of as a risk factor for chronic diseases.

Photobiomodulation has been used in pain and inflammation treatment; nevertheless, a synthesis of its effects and the quality analysis of the studies have never been studied. The goal of this study was to analyze the scientific evidence that currently exists on PBM’s effectiveness when used in the treatment of chronic musculoskeletal pain and inflammation. Secondly, to evaluate the methodological quality of the collected literature.

## 2. Methodology

### 2.1. Study Design

The present study is a literature review on randomized clinical trials (RCTs). In order to achieve a high methodological quality, a methodology characteristic of a systematic review, including the methodological quality of the selected RCTs, was carried out in accordance with the PRISMA criteria for the development and preparation of meta-analysis and systematic reviews [13].

### 2.2. Sources of Information and Search Strategies

The literature search was carried out between March, April, and May 2022 using the following five electronic databases: PubMed, ProQuest, Web of Science, Scopus, and PEDro (see Table 1).

The PICO strategy components were followed to develop the research questions and were based on the following elements:

- Population: patients older than 18 years of age and with inflammation or pain lasting for more than 3 months.
- Intervention: reduction in the symptomology of pain or inflammation after a PBM intervention.
- Comparison: the use or not of PBM.
- Results: effectiveness of the PBM application strategies on chronic pain and inflammation.

#### 2.2.1. PubMed

The search for articles in PubMed used Medical Subject Headings (MeSH), as well as key terms not included in MeSH such as “photobiomodulation”.

The keywords were “Low-level laser therapy”, “metabolism”, “inflammation”, “physiotherapy”, “chronic pain” and “photobiomodulation”.

The Boolean operators were: AND/OR.

The following parameters were used to limit the search strategy of this study:

- Text accessibility: open-access or accessible to the University of Granada, Spain.
- Type of articles: RCTs and observational studies.
- Date of publication: 2017–2022.
- Species: human.
- Language: English.

#### 2.2.2. ProQuest

The keywords “low-level laser therapy”, “metabolism”, “photobiomodulation”, “inflammation”, “physiotherapy” and “chronic pain” were used for the search.

The Boolean operators were: AND/OR.

The following parameters were used to limit the search strategy of this study:

- Text accessibility: open-access or accessible to the University of Granada, Spain.
- Type of articles: RCTs and observational studies.
- Date of publication: 2017–2022.
- Language: English.

#### 2.2.3. Scopus

The keywords used for the search were: “Low-level laser therapy”, “chronic pain”, “photobiomodulation” “physiotherapy, “metabolism” and “inflammation”.

The Boolean operator used was: AND.

The following parameters were used to limit the search strategy of this study:

- Text accessibility: open-access or accessible to the University of Granada, Spain.
- Type of articles: articles.
- Subject: medicine and health professions.
- Date of publication: 2017–2022.

#### 2.2.4. Web of Science

The keywords for the search were: “Low-level laser therapy”, “chronic pain”, “photobiomodulation”, “metabolism”, “physiotherapy” and “inflammation”.

The Boolean operator was: AND.

The following parameters were used to limit the search strategy of this study:

- Text accessibility: open-access or accessible to the University of Granada, Spain.
- Type of articles: articles (RCTs).
- Subject: medicine and health professions.
- Date of publication: 2017–2022.

#### 2.2.5. PEDro

The keywords for the search were: “Low-level laser therapy”, “chronic pain”, “physiotherapy”, “photobiomodulation”, “metabolism” and “inflammation”.

The Boolean operator was: AND.

The following parameters were used to limit the search strategy:

- Text accessibility: open-access or accessible to the University of Granada, Spain.
- Subject: chronic pain.
- Method: RCTs.
- Date of publication: 2017–2022.

### 2.3. Study Selection

#### 2.3.1. Criteria for Inclusion

- Publication between January 2017 and May 2022.
- International publication in English and peer-reviewed.
- Designed as RCTs.
- Access to the complete text available.
- Conducted in humans over the age of 18.
- Suffer from acute pain or chronic pain.
- Suffer from some type of pathology that causes chronic pain.
- Suffer from edema/inflammation.

#### 2.3.2. Exclusion Criteria

- Not meeting the inclusion criteria.
- Repeated in the databases.
- Does not assess chronic pain, inflammation or pathology or symptom prevention.
- Lack of PBMT implementation in people with pain or inflammation.
- No results were shown or no interpretation of the data.

### 2.4. Article Selection

This literature review aimed at focusing on photobiomodulation therapy in general. For this reason, the scope of the search included all areas of low-intensity laser research and the impact it has on various facets within the health fields of medicine and physiotherapy. These studies encompassed the effect of a low-intensity laser on diseases such as fibromyalgia to articles that revealed the part these lasers play in impairments in our temporomandibular joint or teeth. The first searches resulted in 59 eligible studies, which either proved or linked PBM and the benefits it can bring to health regarding the most important areas we wanted to cover in this study.

In order to increase the search quality, more focused searches were carried out on the influence PBM has on chronic pain, at all levels, without neglecting the impact it has on the diseases and injuries in the subjects studied.

Although the search terms were further limited and reflected scientific evidence for PBM in subjects over 18 years of age, studies that did not fall within the scope of our study continued to appear.

Photobiomodulation therapy has been classified as a safe, non-invasive treatment modality. Several possible mechanisms have been attributed to PBM such as an increased thermal threshold, improved blood circulation, increased oxygen consumption by accelerating the redox reaction rate, and increased production of ATP and proinflammatory cytokines [14].

Therefore, the scope of this literature review was reduced to studies that researched any link between PBM and the benefits it can bring to our health, with the primary focus on its influence on pain and inflammation.

### 2.5. Methodological Quality Assessment of the RCTs

The assessment of the methodological quality of the selected RCTs followed the PEDro scale, which was adapted and translated to the Spanish language and allowed the items to be both validated and evaluated.

Each item, excluding the first, raises the overall score by 1 point. Eleven items can be included, resulting in a global score of 0 to 10 points [15].

Subsequently, the Internal Validity Score (IVS) was used to evaluate the internal validity of the RCTs. This scale contains the items that are indicated to be the most representative in ascertaining the internal validity of an article. The selected items are 2, 3, 5, 6, 7, 8, and 9, so the internal validity will have a score of up to 7 points [15].

### 2.6. RCTs Qualitative Synthesis

The score given by the scale determined the quality of the study. Hence, a score of 9–10 points is accepted as having excellent methodological quality, a score of 6–8 points has a good methodological quality, those in the 4–5 point range have a regular quality, and finally, those trials that obtain less than 4 points have a poor methodological quality. Studies that fall within this last range were discarded from this study [15].

The IVS classification of the studies is as follows: studies that receive 6–7 points are given a methodological quality that is high, those with 4–5 points a moderate methodological quality, and 0–3 points have a limited methodological quality [15].

### 2.7. Management of the Identified Literature

The bibliographical references that appear in this review have been managed using Mendeley software.

## 3. Results

This section provides the results found in the selected RCTs.

### 3.1. Study Selection

The searches resulted in 96,751 articles being found in the 5 databases as follows: PubMed, Scopus, PEDro, ProQuest, and Web of Science.

Articles that did not meet the inclusion criteria set out in the “inclusion criteria” section were discarded. The titles and summaries of the found articles were read and the information was summarized to highlight the most relevant aspects, both positive and negative, of this therapy.

**Figure 1:**
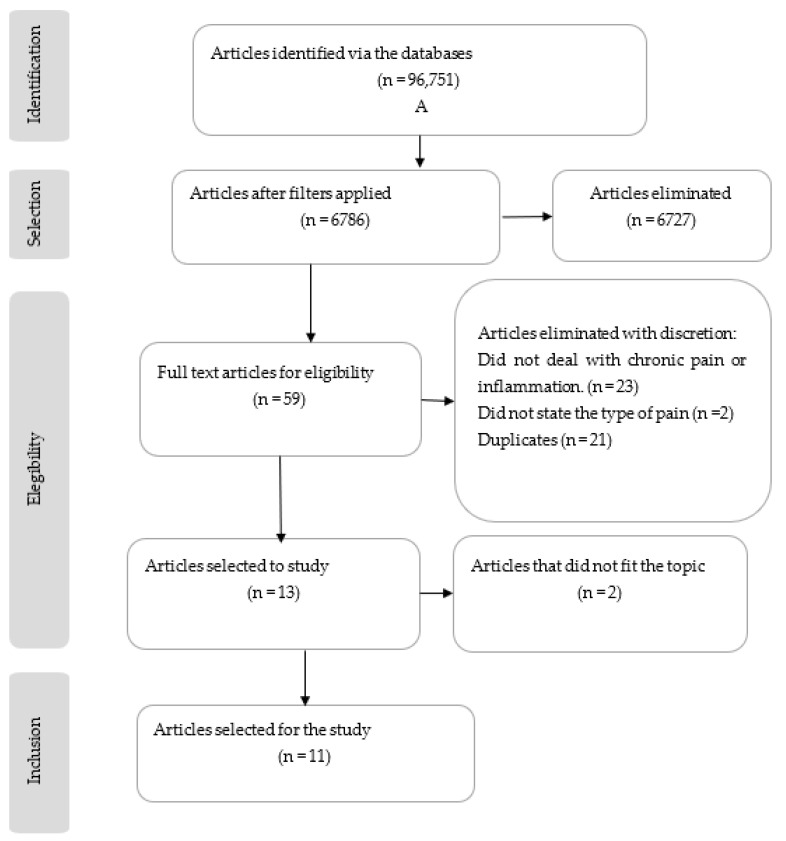
Flow diagram following PRISMA system. Selection and exclusion process for the studies.

The end result of the search and the methodological quality study of the preselected articles was 11 RCTs that met the admission criteria. The RCT selection process is shown in Figure 1 by means of a flowchart in accordance with the guidelines and criteria established in the PRISMA 2009 declaration [13].

### 3.2. Methodological Quality Evaluation

Once the articles had been selected, an evaluation of methodological quality was carried out following the aforementioned scales. The results are offered in Table 2, Table 3 and Table 4.

### 3.3. Study Characteristics

Table 4 shows the main characteristics of the analyzed studies with the most important information being narrowed down to clarify the hypotheses proposed by this study. The type of study, the sample size, the intervention performed, the variables analyzed, and the results obtained, with their respective numerical values, are among the properties found.

### 3.4. Methodological Quality of the Collected Literature

Two scales were used to carry out the methodological quality of the present review. The PEDro scale resulted in eleven articles being selected of which six were given an “excellent” score and the remaining five a “good” score. The IVS resulted in seven articles receiving a “high” score and four were “limited”.

### 3.5. Effects of Photobiomodulation Therapy on Health Improvement and Chronic Pain

The articles in this study were chosen in order to determine the efficacy of PBMT application on subjects with health disorders such as pain or inflammation.

In the subsequent points, the results of the highlighted variables from the selected studies are presented.

#### 3.5.1. Chronic Pain

In this study, 11 articles have been selected that deal with different types of chronic pain.

In the studies by Fernanda R. et al. [18], Leyla K. et al. [19], Mariana M. et al. [20], De Souza R. et al. [21], Shaiane S. et al. [22], Taradaj J. et al. [24], and Fang-Y L. et al. [25] PBMT was applied to patients with chronic pain4

Two of these studies, by Fernanda R, et al. [18] and Fang-Y L. et al. [25], deal with female patients, over the age of 60, with knee osteoarthritis. Both studies revealed significant improvements in the patients, with *p*-values less than 0.05 in all the studied variables related to chronic pain.

On the other hand, we find the studies by Leyla K. et al. [19], Shaiane S. et al. [22], and Taradaj J. et al. [24] which have subjects with nonspecific chronic low back pain. In the first of these, in which there was an equal ratio of men and women and an average age of 49, LLLT produced significant changes in pain and functional status (*p* < 0.001) during the 3-month treatment period, unlike the placebo group that only saw these changes for the first month. The study by Taradaj J. et al. [24] also included subjects of both sexes with an average age of 45. The results showed that the changes produced by PBMT were short-term (3 weeks after therapy), since the 1- and 3-month evaluations resulted in a *p* > 0.05 when the exercises were not continued. The last remaining study is that of Shaiane S. et al. [22], which has a mean age of 34 and includes both male and female participants. The study indicates that PGE2 significantly reduced (*p* = 0.04), therefore, the pain decreased.

The studies by Mariana M. et al. [20] y De Souza R. et al. [21] are comprised of participants with fibromyalgia, and also focus on pain, among other variables. In the first study, the participants are exclusively female, while in the second only the majority are women. The average age of the first study is 35, with 46 being the average age in the second. The study by Mariana M. et al. [20] found that a PBMT session was markedly better than an exercise session, however, no additional benefits were found with a combination of both therapies. In the long term (10 weeks) the combined therapy group and the PBMT group experienced a significant reduction in pain compared to the placebo and the therapeutic exercise groups. The De Souza R. et al. [21] study evaluated the orofacial pain of these FM patients, and the results indicate a significant decrease in pain (*p* = 0.0001) due to the treatment, although with no noticeable difference between the two groups.

#### 3.5.2. Inflammation

In the studies by Gonçalves Langella L. et al. [17], Shaiane S. et al. [22], and Chong R. et al. [19] the fundamental part of the research was to observe the effect of PBMT on the most important biomarkers related to inflammation. The first of these studies observed changes in the serum levels of the proinflammatory cytokines IL-6, IL-8, and TNF-α, in 18 patients after knee arthroplasty surgery, pointing to the fact that PBMT significantly reduced inflammation (*p* < 0.05) when compared to the placebo group. The second of these studies also measured the serum levels of IL-6 and TNF-α in 18 patients with nonspecific chronic low back pain after applying LLLT, but unlike the first, the serum changes in these cytokines did not cause a significant reduction in pain. The last of these studies looked at lipid biomarkers associated with pain and inflammation in 54 people with orthodontic pain and periodontal inflammation. It was noted that the LLLT group had significant improvements in pain 1 h and 24 h later (*p* = 0.02) and in inflammation during the first 24 h.

#### 3.5.3. Acute Pain

Nunes EC. et al. [20] and Gonçalves Langella L. et al. [17] tried to observe how PBMT affects pain but in this case acute pain. The former study, in which groups with a similar male/female ratio participated, used the VPRS and the NPRS since the participants were post-endodontic surgery patients. The study showed that the reduction in pain with laser therapy was significant (*p* < 0.001). The second study also indicates a significant reduction in pain, but this time after knee arthroplasty surgery (*p* < 0.05).

Fernanda T. et al. [26] evaluated temporomandibular pain in a study made up of 41 patients with temporomandibular dysfunction and pain. The groups received PBMT, manual therapy, or a combination of both. The results revealed that there were significant changes in pain reduction (*p* < 0.001), with all managing to reduce it significantly, but this difference did not exist between groups.

### 3.6. Questionnaires and Scales Used in the Articles

A visual analog scale (VAS) is used in the quantitative measurement of pain. It is a linear measurement in which the ends reflect the minimum level at one end and the maximum level of pain at the other. The scale contains a single item that can give a score from 0 to 10. Normally, “no pain” results in the number 0, and “very intense pain/worst imaginable pain “ results in 10 [27].

The Fibromyalgia Impact Questionnaire (FIQ) consists of a three-factor structure with a physical symptoms domain (six items), a functional domain (ten items), and a mental symptoms domain (two items) [28].

Research Diagnostic Criteria (RDC) classify subjects with temporomandibular disorders following a physical diagnosis and their status related to pain disability and psychological functioning [29].

The Roland Morris Questionnaire (RMQ) measures low back pain, consisting of an action list (24 items) that usually affects low back pain [30,31].

Numerical Pain Scale (NPRS): It was developed with the purpose of unidimensionally assessing adult pain. It is an alternative version of the VAS, using a horizontal line, with 11 items, and a score range of 0–10, with 0 being “no pain” and 10 being “more intense pain than can be imagined” [27].

Western Ontario and McMaster Universities Osteoarthritis Index (WOMAC) measures and evaluates pain, functional capacity, and stiffness in osteoarthritic knee and hip diseases. It is made up of 24 items that are divided into three scales. Each item is answered using a verbal scale which has five levels (0–4) [12].

The Beck Anxiety Inventory (BAI): This inventory was designed to assess anxiety in the cognitive and physical domains in the last week before completing the questionnaire. It consists of 21 items and the score can be between 0 and 63. Levels 0 to 7 mean minimal anxiety, 8 to 15 mean mild anxiety, 16 to 25 mean moderate anxiety 16–25, and 26 to 63 mean severe anxiety [32].

## 4. Discussion

The goal of this review was to evaluate the current knowledge on the efficacy of PBM in chronic musculoskeletal pain, acute pain, and inflammation treatment plans. Moreover, to assess the methodological quality of the included studies.

The search and selection process resulted in 11 articles which were all randomized clinical trials.

Of the selected trials, seven dealt with how chronic pain is or is not modified after the application of PBM [16,18,19,20,21,23,33,34], with the VAS being used as the main element for measuring pain.

There are articles [17,18,19] that focus on the impact PBM has on inflammation. The first two studies check its efficacy by measuring the serum levels of the proinflammatory cytokines IL-6, Il-8, and TNF- α. The latter study [19] observed the FGC biomarkers associated with inflammation.

Among the selected studies, there are several that use the VAS to quantitatively measure the changes, and also study the effect of low-intensity laser on patients with acute pain [16,19,20].

In the following sections, we will analyze the impact that PBM has had on different conditions.

### 4.1. Chronic Nonspecific Low Back Pain

Chronic nonspecific low back pain is the most prevalent painful musculoskeletal condition. It is heterogeneous since it can have many causes and diagnoses, but there are very few therapies with solid evidence of efficacy [33].

Low-level light therapy has played a different role in each study. In one study [33] the EG consisted of seven men and thirteen women with an average age of 47 years. After the interventions were carried out and the results evaluated, the subjects were found to have experienced significant improvements in pain and functional state with the improvements lasting for the 3-month treatment period. On the other hand, a study [23], with an average age of 34 years and made up of four men and five women did not find a significant improvement in pain, unlike in the aforementioned study. The methods and parameters of the therapy application were similar, although not identical. In the last of the studies the average age of the sixteen subjects in the EG, eight men and eight women was 45.5. This study concluded that the application of PBM had a significant influence on mobility improvement and hence pain, but only in the short term; the parameters deteriorated at the 1- and 3-month post-therapy analyses where there were no exercises [19].

Knee osteoarthritis

Osteoarthrosis, a very prevalent musculoskeletal condition, is thought to affect 10% of men and 13% of women who are 60 years of age or older. This pathology affects the majority of the affected joints’ tissues and structures, which produces structural and functional changes [3].

The first of the selected studies was made up of two experimental groups, one received US + LLLT while the other received the same with the addition of ET. The age range of the women who made up each group ranged from 61 to 77 years. The results showed a significant increase in pain threshold, and the number of squats in the experimental group when compared to the control group.

Another RCT on PBM was conducted in patients with osteoarthritis. There were 16 patients of both sexes between the ages of 63 and 78 who received PBM three times per week for 4 weeks. Mobility was significantly improved each week compared to the CG. The same occurred with pain threshold and the knee OA severity index [16].

### 4.2. Fibromyalgia

This syndrome is common and is characterized by widespread chronic pain, fatigue, and sleep disorders that severely attack those affected. The etiopathogenesis of this syndrome is not clear, but therapy is available to improve quality of life [35].

In the first of the selected studies with patients suffering from FM, two sets were formed that received the same therapies, namely PBM, ET, and PBM + ET. Set 1 was used to evaluate the therapies in the short term and set 2 in the long term. The age range of 32–42 years old in the two sets was similar. The results determined that the ET group was the only group in set 1 not to significantly improve pain threshold. The PBM + ET group showed no additional benefits in set 1 but did in set 2, which examined the changes over a 10-week period, thus showing that PBM and PBM + ET produced significant improvements in pain threshold. Both combined therapies reduced the number of tender points and had a beneficial role in patients’ anxiety, depression, and fatigue [20].

The second study, unlike the previous one, did not have a CG, but instead had two EGs each comprised of 33 people suffering from FM and with an average age of 46 years. One group received PBM and the other an aesthetic infiltration of lidocaine. The group that received PBM showed a significant decrease in the intensity of orofacial pain, in the same way as the group that received the infiltration. In both groups, all the analyzed study variables improved significantly, although the patients’ perception of well-being was better for the group that received laser irradiation [21].

Current research shows promising short-term results of whole-body PBM on fibromyalgia sufferers, however, the long-term results are still to be explored [6,7].

### 4.3. Temporomandibular Pain and Dysfunction

This disorder is a very common condition experienced mainly by young middle-aged women. The etiology is unknown but the following risk factors seem to be associated with it: bruxism, depression, multiple pain conditions, gender, trauma, and hypermobility [36].

In this study, we incorporated an RCT that verifies the efficacy of photobiomodulation in this condition. It was verified by applying PBM three times a week for a month. The study had no CG but had three EGs receiving, TFBM, TM, or a combination of both. The study had 41 subjects, only 2 of them men. All groups experienced a significant reduction in pain and anxiety. PBM promoted improvements in five conditions, TM in two, and combined therapies in one [17].

### 4.4. Oral Pain and Inflammation

Two selected RCTs tried to report on the impact of PBM on patients with periodontal and endodontic pain and inflammation. The first applied laser therapy to a group of 35 people ranging from 20–37 years old with similar male/female ratios. This article highlights the significant improvement that the therapy produced during the first 24 h when compared to the placebo group [25].

The other group had an EG of 27 people who, after receiving the interventions, were evaluated for subjective pain and the FGC biomarkers associated with pain. The group that received the PBM obtained improvements 1 h and 24 h after treatment. Additionally, subjective pain was also significantly better than in the CG at an interval of 3 days post-application [24].

### 4.5. Post-Surgery Pain and Swelling

Inflammation is known to be the body’s response to an injury to our system. Inflammatory mediators induce pain through the direct activation of nociceptors, hence the two are closely related concepts [37].

One article reviewed the influence of PBM on acute pain and swelling after knee arthroplasty. To accomplish this, nine patients in the EG had an application of PBM. The results revealed a positive and significant improvement in both pain and inflammation post-therapy on five points along the surgical scar [22].

### 4.6. Strengths and Weaknesses of the Study

The present review shows the following strengths:

Good methodological quality and therefore the results are assured by the use of the PEDro scale.

The wide variety of medical conditions presented by the subjects of the selected studies.

The study highlights the lack of studies on this subject and hence the proposal for a novel therapy that can non-invasively address chronic musculoskeletal pain and inflammation.

As weak points there are certain limitations:

Limited RCTs showing the influence of PBM on subjects with inflammation.

A lack of studies that describe the ideal standard parameters according to the type of condition. Since only English studies were included, the extent of the study is reduced.

Heterogeneity of the study subjects, due to the fact that chronic musculoskeletal pain conditions come from different etiologies. Therefore, biomarkers and pain-generating mechanisms may differ.

### 4.7. Prospective

After observing and analyzing the results, it can be seen that the scientific evidence on PBM in relation to chronic pain and inflammation is scarce. Therefore, different lines of research are proposed as follows:

Implementation of RCTs on the efficacy of PBM application strategies in subjects with different kinds of inflammation.

Conduct RCTs on the efficacy of PBM application strategies in subjects with chronic musculoskeletal pain.

Perform RCTs that try to discern standard parameters according to the type of pain or inflammation, in order to move towards a consensus and be able to research the influence of laser therapy under standardized conditions.

Although new evidence analyzing the effects of PBM on sports performance has been carried out [38], studies that show the effects of PBM on elite athletes suffering from pain are still needed.

Finally, studies in subjects below 18 years of age who suffer from acute or chronic pain are necessary.

## 5. Conclusions

The quality of the studies collected on the application of PBM in the treatment of inflammation and chronic pain is generally high, so they have excellent quality in terms of methodology.

The symptoms of pain and chronic pain are influenced by laser irradiation. However, there is a lack of studies to clarify this ability to reduce pain, especially in the long term.

There is not enough evidence on the effects and benefits of PBM on inflammation. Although the studies confirm the positive effects that this therapy has on proinflammatory biomarkers, there are only a few RCTs that verify this, and more research is needed.

## Figures and Tables

**Table 1:** Search strategy in the different databases used.

PubMed	“Low-Level Light Therapy” (MeSH) AND “chronic pain” (MeSH) “Low-level Laser Therapy” (MeSH) OR “photobiomodulation” AND “physiotherapy” “Low-level Light Therapy” (MeSH) AND “inflammation” (MeSH)
ProQuest	“Low-Level Light Therapy” AND “chronic pain” “Low-level Light Therapy” OR “photobiomodulation” AND “physiotherapy” “Low-level Light Therapy” AND “metabolism” “Low-level Light Therapy” AND “inflammation”
Scopus	“Low-level light therapy” AND “chronic pain” “Low-level light therapy” AND “inflammation”
Web of Science	“Low-level light therapy” AND “chronic pain” “Low-level light therapy” AND “inflammation”
PEDro	“Low-level light therapy” AND “chronic pain” “Low-level light therapy” AND “inflammation”

**Table 2:** Study of the methodological quality of the RCTs with the PEDro scale.

Author, Year	1 *	2	3	4	5	6	7	8	9	10	11	Total
Eduardo C. et al., 2020 [16]	1	1	1	1	1	1	0	1	1	1	1	9/10 Excellent
Luciana GL. et al., 2018 [17]	1	1	1	1	1	1	1	1	1	1	1	10/10 Excellent
Fernanda R. et al., 2018 [18]	1	1	0	1	1	0	0	0	0	1	1	6/10 Good
Leyla K. et al., 2020 [19]	1	1	1	1	1	0	0	0	0	1	1	6/10 Good
Mariana M. et al., 2018 [20]	1	1	1	1	1	1	1	1	1	1	1	10/10 Excellent
De Souza R. et al., 2018 [21]	1	1	0	0	0	0	1	1	1	1	1	6/10 Good
Shaiane S. et al., 2021 [22]	1	1	1	1	1	1	1	0	1	1	1	9/10 Excellent
Chong R. et al., 2020 [23]	1	1	1	1	1	1	1	1	1	1	1	10/10 Excellent
Taradaj J. et al., 2019 [24]	1	1	1	1	1	0	1	1	1	1	1	9/10 Excellent
Fang-Yin L. et al., 2020 [25]	1	1	1	1	1	0	1	1	0	1	1	8/10 Good

1. The selection criteria were specified. 2. Subjects were randomly assigned to groups. 3. Allocation was concealed. 4. Groups were similar at baseline regarding the most important prognostic indicators. 5. All subjects were blinded. 6. All therapists who administered the therapy were blinded. 7. All assessors who measured at least one key outcome were blinded. 8. Measures of at least one of the key outcomes were obtained from more than 85% of the subjects initially assigned to the groups. 9. Results were presented for all subjects who received treatment or were allocated to the control group, or where this could not be, data for at least one key outcome was analyzed by “intention to treat”. 10. The results of statistical comparisons between groups were reported for at least one key outcome. 11. The study provides point and variability measures for at least one key outcome. * Non-summation criterion for the PEDro scale [15].

**Table 3:** Internal validity of the selected RCTs.

Author, Year	2	3	5	6	7	8	9	IVS
Eduardo C. et al., 2020 [16]	1	1	1	1	0	1	1	6/7 High
Luciana GL. et al., 2018 [17]	1	1	1	1	1	1	1	7/7 High
Fernanda R. et al., 2018 [18]	1	0	1	1	0	0	0	3/7 Limited
LeylaK. et al., 2020 [19]	1	1	1	0	0	0	0	3/7 Limited
Mariana M. et al., 2018 [20]	1	1	1	1	1	1	1	7/7 High
De Souza R. et al., 2018 [21]	1	0	0	0	1	1	1	4/7 Limited
Shaiane S. et al., 2021 [22]	1	1	1	1	1	0	1	6/7 High
Chong R. et al., 2020 [23]	1	1	1	1	1	1	1	7/7 High
Taradaj J. et al., 2019 [24]	1	1	1	0	1	1	1	6/7 High
Fang-Yin L. et al., 2020 [25]	1	1	1	0	1	1	0	5/7 High
Fernanda T. et al., 2018 [26]	1	0	0	0	1	1	1	4/7 Limited

IVS: Internal Validity Score [15].

**Table 4:** Development of the general characteristics for the selected studies.

Author, Year	Type of Study	Experimental Group	Control Group	Interventions	Variables	Results
Nunes EC. et al., 2020 [16]	Randomized Clinical Trial	35 people with post endodontic surgery pain. Men: 16 Women: 19 Age range: 20.8–37.2 Chronic illness: No Post operative pain: 1.3–2.9 Treatment time: 39.2–54.2	35 people with post endodontic surgery pain. Men: 15 Women: 20 Age range:18.4–42.2 Chronic illness: No Post operative pain: 1.4–3.2 Treatment time: 49.9–39.3	EG: Received PBMT at 4 points (attached to mucosa) after endodontic treatment. CG: Received endodontic treatment and prescribed ibuprofen 600 mg, to be taken every 12 h daily. Received sham PBMT.	Verbal rating scale (VPRS) with ibuprofen. Verbal rating scale (VPRS) with PBMT. Numerical rating scale (NPRS) with ibuprofen. Numerical rating scale (NPRS) with PBMT.	There is a significant decrease in pain in the first 24 h with PBMT compared to the administration of ibuprofen 600mg (*p* < 0.001).
Gonçalves Langella L. et al., 2018 [17]	Randomized Clinical Trial	9 people in the post-surgical period of knee arthroplasty.	9 people in the post-surgical period of knee arthroplasty.	EG: PBMT applied at 5 points along the scar of the operation. CG: Sham PBMT applied at 5 points along the scar of the operation.	Changes in color. Changes in IL-6. Changes in IL-8. Changes in TNF-α.	There was a significant change in the group treated with PBMT, with reduced pain and serum levels of proinflammatory cytokines (*p* < 0.05) (IL-6, IL-8, TNF-α).
Fernanda R. et al., 2018 [18]	Randomized Clinical Trial	Group US + LLLT: 14 Caucasian women with knee osteoarthritis. Age range: 77–67 Group US + ET + LLLT: 14 Caucasian women with knee osteoarthritis. Age range: 71–61	14 Caucasian women with knee osteoarthritis. Age range: 69–61.	EG1 US + LLLT: US and LLLT applied to 5 specific points on the knee. EG2 US + ET + LLLT: US and LLLT applied to 5 specific points on the knee. In addition, performance of guided therapeutic exercise. CG: sham US and LLLT applied to 5 specific points on the knee. Subjects were blinded.	Changes in pain pressure threshold. Changes in the number of squats Radiological Evaluation (K-L Scale)	There was a significant increase in the pain pressure threshold (*p* < 0.01). The number of squats was higher in the experimental groups after treatment when compared to the CG (*p* < 0.0001). There were no radiological findings that differed between any of the groups. (*p* > 0.05).
Leyla K. et al., 2020 [19]	Randomized Clinical Trial	20 people with nonspecific chronic low back pain. Men: 7 Women: 13 Age range: 25–65 Average age: 47	20 people with nonspecific chronic low back pain. Men: 5 Women: 15 Age range: 28–69 Average age: 51	EG: LLLT applied to the joint spaces of the spine (3 points per disc, 30 s each), adjacent paravertebral points, radiating pain zones, and tender and acupuncture points. CG: The same intervention as the experimental group but in the form of sham treatments.	Changes in pain intensity (VAS). Changes in the range of motion of the lumbar spine. Functional status of the patients (according to RMQ). Spinal tenderness.	Significant changes in pain, functional status (*p* < 0.001) and range of motion of the lumbar spine, with these benefits persisting during the 3-month treatment period (*p* < 0.001), unlike in the control group where there were only improvements during the first month. There were no significant differences between groups regarding spinal tenderness.
Mariana M. et al., 2017 [20]	Randomized Clinical Trial	PBMT group: Formed of 2 groups, each containing 20 people with FM. ET group: Formed of 2 groups, each containing 20 people with FM. PBMT + ET group: Formed of 2 groups, each containing 20 people with FM. Set 1: Investigates the immediate effect of a single PBMT/ET session on chronic pain condition Age: 32–38 BMI (Kg/m^2^): 21–31 Set 2: Investigates the long-term effect (10 weeks) on the chronic pain condition and other FM symptoms Age: 38–42 BMI(Kg/m^2^): 23–31	Divided into 2 groups, each containing 20 people with FM. Set 1: Investigates the immediate effect of a single PBMT/ET session on chronic pain condition Age: 32–38 BMI (Kg/m^2^): 21–31 Set 2: Investigates the long-term effect (10 weeks) on the chronic pain condition and other FM symptoms Age: 38–42 BMI (Kg/m^2^): 23–31	EG1 (Set 1): Only one PBMT session was performed. These patients were evaluated at baseline and after 24 h. EG2 (Set 2): PBMT was applied 30 min before each exercise session. Outcome parameters were assessed at baseline (before group draw) and 48 h after the last day of intervention. PBMT: Applied for 300 s at each of 10 sensitive pain points. ET: Consisted of aerobic exercises and stretching 2 times/week for 10 weeks. They also performed TMJ exercises. PBMT + ET: They performed a combination of both protocols.	Changes in pain threshold. Changes in pain intensity (VAS). FIQ score. Sleep score. Mouth opening.	Pain threshold did not significantly improve after an exercise session; however, differences were noticeable with a PBMT session. Set 2 of the PBMT group showed similar results to Set 1. In Set 1, no additional benefits of the PBMT + ET combination were detected. The PBMT and PBMT + ET groups showed significant pain reduction versus the ET group and CG (Set 2). Furthermore, both combined therapies reduced the number of tender points. PBMT + ET had a beneficial role in anxiety, depression, and fatigue.
De Souza R. et al., 2018 [21]	Randomized Clinical Trial	66 people with FM. Age Range: 57.05–35.23 Average age: 46.14 (62 women and 4 men) Group A: 33 volunteers with FM and chronic pain. Group B: 33 volunteers with FM and chronic pain.	None.	2 sessions/week for 6 weeks: Group A: GaAIAs diode laser irradiation for 40 s at each selected point. 780 nm wavelength. Power: 50 mW. Energy: 2 J. Point-skin distance: 1 cm. 1 session/week for 4 weeks: Group B: Anesthetic injection of 2% lidocaine without vasoconstrictor. Volume: 0.5 mL at each tender point. Stretching after infiltrations.	Intensity of orofacial pain (VAS). Tenderness in the facial muscles. Perception of efficacy and well-being of both treatments.	Significant decrease in pain (*p* = 0.0001), although without any noticeable difference between groups. There was a decrease in pain intensity in tender points with both treatments. All the muscles analyzed had a response except for the temporal post (*p* < 0.05). The perception of the patients and their well-being improved in both groups with a slight difference in favor of Group A.
Shaiane S. et al., 2021 [22]	Randomized Clinical Trial	9 people with chronic nonspecific low back pain. Women: 5 Men: 4 Age range: 20.51–47.05 Average age: 33.78	9 people with chronic nonspecific low back pain. Women: 6 Men: 3 Age range: 19.63–44.23 Average age: 31.44	EG: A single PBMT session was applied with the following parameters: Area: 4 cm^2^. Frequency: 3000Hz. Time: 3 min at each sensitive point. Total energy radiated at each point (between the spinous processes of T11 and T12, L2 and L3, L5 and S1 and in the same direction but laterally, 3 sites to the left and another 3 to the right): 24.30 J. CG: Received the same therapy, but in a simulated way, in a single session.	Changes in PGE2. Pain intensity. Changes in IL-6. Changes in TNF-α.	With PBMT, PGE2 decreased significantly (*p* = 0.04) compared to the placebo group. The same did not happen with pain intensity, IL-6 or TNF-α, which did not show significant changes in relation to the CG.
Chong R. et al., 2020 [23]	Randomized Clinical Trial	27 people with orthodontic pain and periodontal inflammation.	27 people with orthodontic pain and periodontal inflammation.	EG: For 6 months the use of PBMT on orthodontic pain and periodontal inflammation is examined. During the first 2 months the volunteers had braces/buccal tubes in the maxillary and/or mandibular dental arches, followed by thermal NiTi archwire treatment in the 3rd month. The teeth in the middle of the dental arch were treated with LLLT. With a quadrangular probe, the region was covered from the central incisor to the first molar. The probe was first directed at the level of the braces and the buccal gingival margin and then moved to the level of the buccal alveolar mucosa. Wavelength: 940 nm. Output power: 800 mW. Irradiation duration: 30 s. CG: Received the same treatment as the experimental group but with a sham LLLT.	Subjective pain assessment. Clinical periodontal status and supragingival bacterial load. FGC biomarkers associated with pain and inflammation.	In the comparison between groups, regarding subjective pain, the side irradiated with LLLT experienced less pain, but it was only significant at the 6- and 24 h intervals (*p* = 0.01) and marginally significant at the 3-day interval (*p* = 0.03). No differences were found between groups (*p* > 0.05). regarding the clinical periodontal status and the bacterial load. The LLLT group had significant improvements in both pain 1 h and 24 h later (*p* = 0.02) and gingival inflammation during the first 24 h (*p* = 0.00).
Taradaj J. et al., 2019 [24]	Randomized Clinical Trial	TLAI group: 18 people with nonspecific chronic low back pain. Men: 10 Women: 8 Age range: 29–58. Average age: 44 LLLT group: 16 people with nonspecific chronic low back pain. Men: 8 Women: 8 Age range: 29–53 Average age: 45.50	TLAI group: 17 people with nonspecific chronic low back pain. Men: 9 Women: 8 Age range: 26–51. Average age: 45 LLLT group: 17 people with nonspecific chronic low back pain. Men: 9 Women: 8 Age range: 22–76 Average age: 52	EG: TLAI group: Received 15 daily high-intensity laser irradiations for 3 weeks. Area: 6 × 5 cm lumbar area with a 30 cm^2^ punctual applicator. Wavelength: 1064nm. Energy: 60 J/cm^2^. Duration: 10 min. LLLT group: Received 15 daily high-intensity laser irradiations for 3 weeks. Area: Paraspinal region of the lower back. Wavelength: 785nm. Energy: 8 J/cm^2^. Duration: 8 min. CG: The placebo TLAI group and the placebo LLLT group both received sham irradiation treatments of their respective types of lasers.	Roll path. Roll path along the Y axis. Roll path along the X axis. Average speed. Average frequency. Rolling area results. Tests are performed with eyes closed and eyes open.	The only non-significant changes were seen in the average frequency (*p* = 0.77 (Open Eyes); *p* = 0.68 (Closed Eyes)). Improvements did occur but were only significant in the short term (after 3 weeks of therapy). Analysis 1 and 3 months after therapy, without continuing stabilization exercises, showed the parameters to deteriorate over time. Therefore, the changes are observed over a short period of time.
Fang-Yin L. et al., 2020 [25]	Randomized Clinical Trial	16 people with knee osteoarthritis. Age range: 77.42–63.64.	17 people with knee osteoarthritis. Age range: 76.64–62.82.	EG: Treated with LLLT 3 times a week for 4 weeks at points SP9, SP10 and EX.-LE2 on the knee. Wavelength: 780 nm (P: 50 mW) and 830 nm (P: 30 mW). Duration: 15 min CG: Receives the same treatment as the EG but in a simulated way.	Subjective pain assessment (VAS) (Static and in motion). Changes in the pain pressure threshold (over the goosefoot tendon). Knee OA severity index (Lequesne index).	For the EG, the conscious VAS to move the knees decreased each week, while in the CG it did not decrease significantly. The same thing happened with the knee at rest (*p* < 0.01, *p* < 0.0001). The pain pressure threshold improved significantly each week in the EG, while for the CG this threshold did not improve effectively (*p* < 0.001, *p* < 0.0001). The OA severity index also improved significantly for the EG (*p* < 0.0001, *p* < 0.001)
Fernanda T. et al., 2018 [26]	Randomized Clinical Trial	Groups made up of 41 patients with temporomandibular pain and dysfunction (TMD). PBMT group: 14 patients. Women: 14 Age range: 30–61.4 MT group: 13 patients. Men: 1 Women: 12 Age Range: 20.9–61.5 Combined therapies (CTs) group: 14 patients. Men:1 Women: 13 Age range: 32.1–61.9.	None.	PBMT group: PBMT applied 3 times a week for 4 weeks. Wavelength: 808 nm. Dot size: 0.03 cm^2^. Power: 100 mW. Irradiation: 133 J/cm^2^. Irradiation time: 40 sec/point (12 points). MT group: Received 3 weekly session on intra- and extra- oral masticatory muscles (temporal, masseter, medial pterygoid on both sides) and TMJ for 4 weeks. CTs group: They underwent the MT and PBMT protocols 3 times a week for 4 weeks.	Pain assessment (VAS). Beck anxiety inventory (BAI). TMD classification. Assessment of psychosocial aspects.	All subjects experienced a significant reduction in pain (*p* < 0.001) although not between groups. All groups showed a reduction in anxiety (PBMT: *p* = 0.02; MT: 0.03; CTs: *p* < 0.001). The classification of the TMD, based on Axis I, revealed that all patients were diagnosed with GI and GIII and presented a combination of both myogenic aspects. MT promotes improvement in 2 impairments, PBMT in 5 and CTs in 1 (*p* < 0.001). The evaluation of psychosocial aspects, comparing baseline and follow-up in all treatment groups, revealed that the treatment did not modify the intensity of chronic pain (*p* > 0.05).

## Data Availability

All data associated with this study are present in the paper. All requests for other materials will be reviewed by the corresponding author to verify whether the request is subject to any intellectual property or confidentiality obligations.
